# Comparison of COVID-19 Rates Among In-Person and Virtual Attendees of a National Surgical Society Meeting in the US

**DOI:** 10.1001/jamanetworkopen.2022.30300

**Published:** 2022-09-07

**Authors:** Casey M. Silver, Rachel H. Joung, Melanie S. Morris, Kasper S. Wang, Amir A. Ghaferi, Karl Y. Bilimoria, Callisia N. Clarke

**Affiliations:** 1 Surgical Outcomes and Quality Improvement Center, Department of Surgery, Northwestern University Feinberg School of Medicine, Chicago, Illinois; 2 Department of Surgery, University of Alabama at Birmingham; 3 Department of Surgery, Children’s Hospital Los Angeles, Los Angeles, California; 4 Department of Surgery, University of Michigan, Ann Arbor; 5 Division of Oncology, Department of Surgery, Medical College of Wisconsin, Milwaukee

## Abstract

This survey study compares the COVID-19 case rates among in-person and virtual attendees of a large surgical society meeting held in the US during the Omicron variant surge.

## Introduction

Many professional societies have resumed in-person meetings after canceling or adopting virtual formats during the COVID-19 pandemic. However, rates of viral transmission at such meetings remain unknown. Limited research has suggested increased case numbers after general mass gatherings; attendees of medical meetings are at increased risk of occupational exposure, more adherent to mask guidelines, and vaccinated at a higher rates.^1,2,3^ Reports of cases after in-person attendance have been anecdotal, small, or unpublished.^4^

This survey study compares rates of COVID-19 positivity between in-person and virtual attendees of a large professional society meeting held during the Omicron surge. We hypothesized that stringent precautionary measures would be associated with reduced rates of transmission.

## Methods

This cross-sectional survey study included participants of the Academic Surgical Congress (ASC), one of the largest surgical society meetings in the US. This study followed the American Association for Public Opinion Research (AAPOR) reporting guideline and was deemed exempt research by the Northwestern University Institutional Review Board because data were derived from anonymous surveys.

The ASC was held in Orlando, Florida, on February 1 to 3, 2022. Meeting registrants could participate in person or virtually. Measures to prevent COVID-19 transmission included encouraging self-testing, mandatory vaccination and masking, and outdoor serving of food and beverages (eMethods 1 in the Supplement). Registrants were recruited to participate in a survey assessing COVID-19 testing and symptoms in the 7 days after the meeting (eMethods 2 in the Supplement). Differences in positivity rates between in-person and virtual attendees were evaluated using χ^2^ tests. Data were analyzed with Stata MP, version 17.0 (StataCorp LLC). Two-sided *P* < .05 indicated statistical significance.

## Results

Among 1617 meeting registrants, 681 responded to the survey (42.1%), including 187 students (27.4%), 234 trainees (34.3%), and 226 attending physicians (33.2%). Among respondents, 135 (19.8%) attended virtually and 546 (80.2%) attended in person (Table). Positive test results before the meeting prompted 6 respondents (4.4%) to attend virtually. All in-person participants were fully vaccinated, and 500 (91.6%) had received a booster.

**Table.** Characteristics of In-Person and Virtual Attendees of a Professional Society Meeting^a^

Characteristic	Attendee type (N = 681)	
	Virtual	In-person
Total	135 (19.8)	546 (80.2)
Level of training		
Medical student	52 (38.5)	135 (24.7)
Trainee	59 (43.7)	175 (32.0)
Attending physician	16 (11.9)	210 (38.5)
Other	8 (5.9)	26 (4.8)
Abstract accepted		
Yes	131 (97.0)	458 (83.9)
No	4 (3.0)	88 (16.1)

^a^ All data are presented as No. (%) of attendees.

Positive COVID-19 test results within 7 days of the meeting were reported by 10 in-person (1.8%) and 2 virtual (1.5%) attendees; differences in rates of positivity were not statistically significant (*P* = .83). The most common reason for testing was “I wished to ensure that I had not contracted COVID-19” (86 [69.3%]). Four attendees reported developing symptoms but were not tested. All COVID-19–positive attendees had received a booster. Missed work was reported by 7 of 10 COVID-19–positive attendees (mean [SD], 4.8 [2.7] days), and there were no hospitalizations.

## Discussion

Professional societies have resumed in-person meetings. As new variants arise, it is important to continue to evaluate the risk of COVID-19 exposure. The ASC met just after the peak of the Omicron variant surge.^5^ Conference organizers rapidly adapted planned safety measures to account for increased variant transmissibility and case numbers.^6^ Although most registrants attended the meeting in person, rates of COVID-19 positivity were low and equivalent among in-person and virtual attendees. These data suggest that among highly vaccinated clinicians with high risk of occupational exposure, cautious strategies to mitigate COVID-19 transmission during a surge were effective, and in-person meeting attendance posed no greater risk than professional hazards.

The self-reported data are a limitation of this survey study, because response bias may have limited reporting of a positive test result. Additionally, we were unable to capture cases without testing, although few attendees reported development of symptoms. Although COVID-19–positive attendees may have contracted the virus while traveling, it is important to note that transmission was still related to meeting attendance. Societies should continue to modify preventive strategies and evaluate safety measures so that meetings may be conducted as safely as possible to prevent viral transmission.

## Notes

Supplement. **eMethods 1.** Measures Taken at the 2022 Academic Surgical Congress to Prevent the Transmission of COVID-19 **eMethods 2.** Survey Instrument
