# Supplementary material for: Comparison of COVID-19 Rates Among In-Person and Virtual Attendees of a National Surgical Society Meeting in the US
Source: JAMA Netw Open. 2022 Sep 7;5(9):e2230300. doi: 10.1001/jamanetworkopen.2022.30300 (PMC9453540; doi:10.1001/jamanetworkopen.2022.30300)
Supplement: Supplement. — eMethods 1. Measures Taken at the 2022 Academic Surgical Congress to Prevent the Transmission of COVID-19 eMethods 2. Survey Instrument [file jamanetwopen-e2230300-s001.pdf]

Supplementary Online Content

Silver CM, Joung RH, Morris MS, et al. Comparison of COVID-19 rates among in-person and virtual attendees of a national surgical society meeting in the US. *JAMA Netw Open*. 2022;5(9):e2230300. doi:10.1001/jamanetworkopen.2022.30300

eMethods 1. Measures Taken at the 2022 Academic Surgical Congress to Prevent the Transmission of COVID-19
eMethods 2. Survey Instrument

This supplementary material has been provided by the authors to give readers additional information about their work.

© 2022 Silver CM et al. *JAMA Network Open*.

eMethods 1. Measures Taken at the 2022 Academic Surgical Congress to Prevent the Transmission of COVID-19

a) An advisory board was formed to examine evidence on viral transmission and make recommendations to the program committee.
b) Information and recommendations were widely disseminated as a meeting checklist for attendees via email and social media prior to the meeting.
c) In-person attendees were strongly encouraged to self-test prior to traveling to the meeting to ensure a negative result.
d) Registrants were barred from in-person attendance if they tested positive for COVID-19 within 14 days of the meeting or developed symptoms of COVID-19 prior to arriving for the meeting.
e) Upon registration, participants were required to provide proof of full vaccination, which was defined as two weeks after completing the second dose of a two-dose vaccine or two weeks after completing the first dose of a one-dose vaccine. Booster vaccinations were encouraged though not required. Those claiming vaccination exemptions for medical or religious reasons needed to show a negative COVID test (antigen or PCR) taken within 72 hours of arrival at the meeting.
f) Attendees were required to wear masks during all meeting events, and only N95, KN95, and surgical masks were acceptable. KN95 masks were made available at the meeting.
g) The main conference rooms incorporated spaced seating, and participants were advised to leave space between seats in smaller parallel sessions.
h) All food and drinks were served outdoors, and participants were prohibited from eating or drinking in meeting spaces.
i) Social and networking events were similarly held outdoors.
j) Sanitization stations were placed throughout the venue.
k) Antigen tests were available by request on-site for those who developed symptoms at the meeting, and local resources for PCR testing and medical care were outlined.

© 2022 Silver CM et al. *JAMA Network Open*.

eMethods 2. Survey Instrument

1. What is your level of training or role at the conference?
    a. Medical student
    b. Resident
    c. Fellow
    d. Attending
    e. Conference staff
    f. Other

2. Did you have an abstract accepted for presentation at the conference?
    a. Yes
    b. No

3. Did you attend the meeting virtually or in-person?
    a. Virtually
       Proceed to virtual participant questions
    b. In-person
       Proceed to in-person participant questions

VIRTUAL PARTICIPANTS

4. Why did you attend the meeting virtually? Check all that apply.
    a. I was concerned about traveling during the COVID pandemic
    b. I tested positive for COVID-19 prior to the meeting
    c. I developed symptoms concerning for COVID-19 prior to the meeting
    d. My institution had imposed travel restrictions
    e. I had other obligations
    f. My travel was limited by weather
    g. Other

   End of survey

IN-PERSON PARTICIPANTS

4. At the time of the meeting, were you fully vaccinated against COVID-19? "Fully vaccinated" was defined as: two weeks after completing the second dose of a two-dose vaccine OR two weeks after completing the first dose of a one-dose vaccine.
    a. Yes
    b. No

5. At the time of the meeting, had you received a COVID-19 booster?
    a. Yes
    b. No

6. At the time of the meeting, had you ever had a COVID-19 infection?
    a. Yes
       When did you have COVID? Check all that apply.
        a. Early in the pandemic - 2020
        b. January 2021 to November 2021
        c. Recently – December 2021 to present
    b. No

© 2022 Silver CM et al. *JAMA Network Open*.

7. Within 7 days of returning home from the meeting, were you tested for COVID-19?
    a. Yes
       Proceed to question 9
    b. No
       Proceed to question 8

8. Within 7 days of returning home from the meeting, did you develop COVID-like symptoms (sore throat, cough, shortness of breath, fever or chills, fatigue, body aches, headache, new loss of taste or smell, congestion, nausea or vomiting, diarrhea)?
    a. Yes
       Proceed to question 11
    b. No
       End of survey

9. Why were you tested for COVID-19? Check all that apply.
    a. I was symptomatic (sore throat, cough, shortness of breath, fever or chills, fatigue, body aches, headache, new loss of taste or smell, congestion, nausea or vomiting, diarrhea)
    b. I wished to ensure that I had not contracted COVID-19 while traveling
    c. I had a known positive contact at the meeting
    d. I had a known positive contact outside of the meeting
    e. Other

10. Did you test positive for COVID-19?
    a. Yes
       Proceed to question 11
    b. No
       End of survey

11. How did you travel to the meeting?
    a. Air
    b. Car
    c. Train

12. Did you go to the emergency room?
    a. Yes
       Proceed to question 13
    b. No
       Proceed to question 17

13. Were you hospitalized?
    a. Yes
       Proceed to question 14
    b. No
       Proceed to question 17

14. Were you admitted to the floor or an ICU?
    a. Floor
        15. Did you require supplemental oxygen?
            1. Yes
            2. No
    b. ICU
        15. Were you ventilated?
            1. Yes
            2. No

© 2022 Silver CM et al. *JAMA Network Open*.

16. How many days were you hospitalized?
    1-10+, or "I continue to be hospitalized"

17. Did you miss work due to your illness?
    a. Yes
        How many days of work did you miss?
            1-10+, or "I have not yet returned to work"
    b. No

© 2022 Silver CM et al. *JAMA Network Open*.
